# Participants’ perceptions of the preconception phase of the *Bukhali* trial for young women in Soweto, South Africa: A qualitative study

**DOI:** 10.1177/17455057261469051

**Published:** 2026-08-25

**Authors:** Catherine E. Draper, Nosibusiso Tshetu, Nokuthula Nkosi, Stephen J. Lye, Shane A. Norris

**Affiliations:** 1 SAMRC Developmental Pathways for Health Research Unit, Department of Paediatrics, Faculty of Health Sciences, 37708 University of the Witwatersrand, Johannesburg, South Africa; 2 90755 Lunenfeld-Tanenbaum Research Institute, Sinai Health, Toronto, Canada; 3 Departments of Obstetrics and Gynecology, Physiology and Medicine, 7938 University of Toronto, Toronto, ON, Canada; 4 School of Human Development and Health, 218266 University of Southampton, Southampton, UK

**Keywords:** preconception health, process evaluation

## Abstract

**Background:**

The preconception phase of the Healthy Life Trajectories Initiative *Bukhali* randomised controlled trial has been completed with young women in Soweto, South Africa. Given the gap in preconception care and interventions in low- and middle-income countries, and low levels of awareness about preconception health as a concept, it is important to understand participants’ perceptions of this phase of the trial.

**Objectives:**

The aim of this study was to report on trial participants’ perceptions of the preconception phase of the trial, with a focus on its acceptability.

**Design:**

This qualitative, descriptive study formed part of the *Bukhali* process evaluation.

**Methods:**

Participants were purposively selected from the intervention (n=22) and control (n=17) arms of the *Bukhali* trial. Eight semi-structured focus groups were conducted with participants (two rounds of focus groups with same participants, including some replacements). A codebook thematic approach was taken to the descriptive process of data analysis.

**Results:**

The main themes identified were perceptions of preconception and pregnancy planning, perceived impact of the trial, motivation for staying involved in the trial, agency, and suggestions for preconception services. The trial was viewed as acceptable by women participating in both the intervention and control arms of the trial, but preconception remains an unfamiliar term. Terms such as pregnancy planning, family planning, and ‘preventing’ added to the confusion around the framing of health promotion for young women who are not pregnant. Participants preferred a longer intervention or service, and wanted this service to be delivered in a similar way to the *Bukhali* trial, not in clinics by nurses.

**Conclusion:**

Addressing preconception health with young women in Soweto should be done in a way that is contextually relevant, and that over time, contributes to their learning and development, benefitting their physical and mental health, irrespective of their pregnancy plans or intentions.

## Introduction

Since the World Health Organization released their guidance on preconception care in 2013,^1^ there has been increasing attention on the importance of preconception health.^2–5^ However, the implementation of preconception care has been suboptimal, and the evidence of the effectiveness of preconception interventions remains scarce.^6,7^ This is especially true in low- and middle-income countries.^8,9^ In Africa, despite women facing numerous risks to preconception health,^10^ implementation of preconception care is low and faces numerous barriers (e.g. lack of policy, limited awareness, inconsistent service provision).^11^ Furthermore, utilisation of preconception care has been found to be low in African countries, and this has been associated with limited knowledge of preconception care, both amongst women and in healthcare workers.^12–15^ Limited knowledge and awareness about preconception care has been found in other studies in Ethiopia^16^ and South Africa (for women and healthcare workers),^17–19^ and studies in South Africa have also highlighted implementation challenges and low utilisation of preconception care.^18–20^

There have recently been critiques of how preconception care has been framed, in terms of limiting women’s needs to those that relate to their capacity to become pregnant and/or their needs as mothers, conflating women’s health and maternal health.^7,21,22^ This has prompted discussions around how preconception care – and health – are conceptualised across diverse contexts, moving beyond individualised approaches to take into consideration the range of social and structural factors that influence behaviours and choices relating to conception and preconception care.^7,22^ Aligning with these critiques has been a call to view preconception through more of an equity lens to draw attention to the social and structural determinants of health, prioritise women’s autonomy around their reproductive health, and avoid risks and harms to women.^22,23^

While the preconception literature from South Africa does not specifically contradict the suggested reframing, it does appear that the traditional conceptualisation of preconception prevails. There also seems to be little exploration of how the features of preconception health apply to women of childbearing age more generally. One South African study highlighted the need for preconception care to be empowering for women, and explored awareness and perceptions of a ‘reproductive life plan’, which resonated with women and healthcare workers.^17^ However, a reproductive life plan is a concept developed and used in high-income countries such as the United States^24^ and Sweden,^25^ and may not consider the structural barriers that women face to health and bodily autonomy in South Africa. The notion of planning implies a level of stability that enables women to plan, as well as some degree of agency around reproductive health. Given the scourge of gender-based violence in South Africa,^26^ alongside a host of socioeconomic challenges and persisting inequities, neither of these can be taken for granted in this context.

### Healthy life trajectories initiative – *Bukhali* trial

The Healthy Life Trajectories Initiative (HeLTI) is an international consortium developed in partnership with the World Health Organization (WHO) in Canada, India, China, and South Africa. HeLTI hypothesises that an integrated complex intervention, comprising a continuum of care from preconception, through pregnancy, infancy and early childhood will promote young women’s physical and mental health, in order to establish healthier trajectories for themselves and future children. For HeLTI SA, the *Bukhali* randomised controlled trial is being conducted with 18–28-year-old women in Soweto,^27^ and the preconception phase of the trial (phase 1) has been completed; the evaluation of this phase of the trial is underway. The *Bukhali* intervention is a health promotion intervention delivered by trained community health workers, referred to as ‘Health Helpers’. These individuals conduct risk screening referral and management support, provide health literacy support and multi-micronutrient supplementation, and support health behaviour change using Healthy Conversation Skills.^28,29^

The preconception phase of the intervention was 18 months long, if a participant did not become pregnant; if she did become pregnant at any point in the 18 months, she transitioned to the pregnancy phase. Sessions were delivered monthly either in-person or telephonic sessions, and topics covered various aspects of physical and mental health.^30^ Although the *Bukhali* intervention was developed as a preconception intervention with the potential of downstream effects for young women and their future offspring (if they became pregnant), the intervention was not specifically framed to participants as a preconception intervention. This was informed by formative work for the trial which highlighted the need to frame the intervention – and trial more generally – in terms of young women’s health, not with the intention of them becoming pregnant.

For the control arm, standard of care ‘plus’ was delivered telephonically by call centre agents, covering life skill related topics in order to keep control participants engaged through the provision of information on topics believed to be of value to young women in the Soweto context. This decision was based on feedback from participants, community members and stakeholders in the formative work prior to the trial which indicated that standard of care through the public health system would not be sufficient to engage participants over 18 months, and certainly not over the full length of the trial (5-6 years if they became pregnant), given the longevity of the project (originally planned for 10 years).^30^ Although the topics covered in the control arm were explicitly not health or nutrition related, there was an ethical commitment to provide control participants basic feedback on their results of health testing conducted at baseline, and that participants scoring in high risk categories (e.g. hypertensive) would receive the appropriate referral within the public health system.

The *Bukhali* trial process evaluation^30^ draws on the UK MRC guidance on process evaluation,^31^ and is grounded in an understanding of the contextual realities and lived experiences of the women participating in the trial. Thus far, the process evaluation has explored trial implementation,^32–35^ participants’ perceptions and experiences (regarding health behaviours,^36^ supplementation,^37^ social support,^38^ and decisions to terminate a pregnancy^39^), retention,^40^ and HHs’ perspectives.^41,42^ Qualitative formative work for the *Bukhali* trial, as well as earlier process evaluation work highlighted low levels of health literacy amongst young women in Soweto,^32,35,37,43^ specifically drawing attention to the ‘preconception knowledge gap’.^44^ Over the course of the trial, we have interrogated the term ‘preconception’ and its meaning for trial participants, while exploring other potential terms that would make sense in the multilingual environment of Soweto.

Our reservations about the appropriateness of the term preconception notwithstanding, as part of the process evaluation of the preconception phase of the *Bukhali* trial we were interested in examining the extent to which this preconception knowledge gap has been addressed, from the perspective of participants. While some process evaluation findings with participants in the infancy phase of the trial have been promising in terms of improvements in health literacy,^40^ investigating this specifically in the preconception phase was seen to be important. In addition, health services for young women in South Africa have not previously been framed as ‘preconception health’. It is thus helpful to explore how terminology relating to this phase is understood by *Bukhali* participants to help contextualise their perceptions, and to find out what health services they think should be offered for young women in this period – and for young women more generally.

The aim of this study was therefore to report on participants’ perceptions of the preconception phase of the trial, with a focus on its acceptability. This included exploring the terminology mentioned above, as well as participants’ suggestions for health services.

## Methods

### Study design

This qualitative, descriptive study formed part of the *Bukhali* process evaluation,^30^ with a focus on the preconception phase of the trial. The UK MRC guidance on process evaluation,^31^ and its theoretical underpinnings informed the methodological orientation of the study. Other process evaluation data (quantitative and qualitative) on the perspectives and performance of trial staff, and the fidelity of the intervention are being reported elsewhere. A completed COREQ checklist^45^ is provided as supplementary material. Based on the experience of the *Bukhali* trial process evaluation team and their interaction with trial participants, focus groups were agreed to be the best method for data collection to achieve this study’s aims.

### Setting and participants

The *Bukhali* trial and process evaluation are being conducted at the research site, located at the Chris Hani Baragwanath Academic Hospital in Soweto. Soweto is a predominantly low-income, densely populated, urban setting in Johannesburg, and young women face multiple risks to their physical and mental health.^30^ Participants were purposively sampled from the intervention (n=22) and control (n=17) arms of the trial, and were contacted telephonically by the process evaluation team to invite them to participate. To be eligible for selection, participants should have recently exited or were about to exit the preconception phase of the *Bukhali* trial, and should not have become pregnant during the trial. Additional selection considerations included: participants who were active throughout their 18 months in the trial and consistently attended their visits, participants from different geographical areas in Soweto to capture variation in experiences, participants who demonstrated good communication and engagement throughout the study, and participants who had a good relationship with trial staff. Participants ranged in age from 19 – 30 years.

### Data collection

A total of eight semi-structured focus groups were conducted with trial participants in the intervention and control arms, facilitated by NT and NN, female staff members on the *Bukhali* trial process evaluation, with more than five years’ experience conducting qualitative research. NT (Project Coordinator) has a Bachelor of Arts (Honours) in Women’s and Gender Studies, and NN (Research assistant) has a MA in Social Work and a MA in Development Studies. Both of these individuals would have been known to participants as members of the *Bukhali* trial process evaluation team. Participants would have been familiar with the research team being interested in their perceptions and experiences as trial participants, and that the data being collected was being used for research purposes. The reasons and interests in the research topic were stated at the beginning of the focus group discussions, and their role in the trial was clarified.

The duration of this study was eight months. A first round of four focus groups was conducted in March 2024 (two intervention, n=15; two control, n=13), and the same participants were invited to participate in a second round of focus groups in October 2024 to follow up on certain topics. Specifically, the second round of focus groups provided an opportunity to present a summary of the key findings from the firsts round of focus groups, and to further probe participants’ recommendations for a health programme targeting young women (e.g. duration, intensity, content, and who should deliver the sessions).

Fourteen participants were not able to participate in the second round for various reasons, so an additional 11 participants were recruited (n=7 intervention, n=4 control) to replace them. Reasons for not being able to participate in the second round of focus groups included relocation (making it more difficult to get to the research site), and work or study commitments. There were also challenges with contacting some participants, which have been previously documented as part of the process evaluation.^40,46^

Although transcripts were not made available for participants, the follow-up focus groups gave facilitators an opportunity to provide some feedback on the previous focus group findings (summarised separately for intervention and control groups), and to explore in more detail potential intervention strategies (or services) for preconception. The discussions ranged in length between 1 hour 44 minutes and 3 hours and 13 minutes. In the debrief discussion after data collection was complete, NT, NN and CD considered the likelihood of any new information emerging from further discussions with trial participants. It was agreed that this was unlikely, given the length of the focus group discussions and the engagement of the participants in these discussions, and that saturation had therefore been reached. However, we would state that achieving data saturation was not an explicit aim of this study.

Focus groups were conducted in a private, quiet and comfortable room at the research site, since this was familiar to participants, and considered to be a safe environment by them. This venue provided a conducive space for participants to speak openly and thus had a positive impact on the data collection process. Refreshments were provided for all groups, and participants’ transport costs were reimbursed. Focus group discussion guides were developed by co-authors (included as supplementary material). The interview guide was not pilot tested, but facilitators (NT and NN) debriefed after the first focus group with CD to discuss any changes that needed to be made to the questions. The focus groups were facilitated by NT and NN; while the discussion guides were developed in English, facilitators were able to converse in local languages when necessary. The discussions were audio recorded, and translated into English and transcribed verbatim. Field notes were taken by the facilitator not asking the questions.

We acknowledge that our training, roles, lived experiences, identities, and values have influenced the research process. Positionality statements for all authors are included in the supplementary material. Table 1 provides further details regarding trustworthiness in qualitative research, as per Lincoln and Guba’s criteria,^47^ in relation to this study.

**Table 1.** Trustworthiness criteria.

Credibility	The focus group facilitators, as members of the *Bukhali* trial process evaluation team, have been engaged with trial participants over a number of years, and have gained valuable insights into the lived experiences and contextual realities of participants. This has enabled them to build rapport with participants, and create an environment of trust during focus group discussions. Apart from acknowledging positionality, the process evaluation team (CD, NT and NN) have engaged in robust discussions regarding their personal biases and preconceptions regarding the topics investigated as part of the process evaluation team, and have brought these considerations to the development of the discussion guide questions. While triangulation of data sources was not used in this particular study, this study forms part of a broader set of studies conducted as part of the process evaluation that have included focus groups, individual in-depth interviews, and observation.
Transferability	The location of the research has been described, and other studies referenced, to help characterise the lived experiences and contextual realities of participants. Participants were purposively sampled to obtain a range of experiences, but also to ensure engagement within discussions.
Dependability	The research process has been documented, and while certain contextual adaptations may be necessary, detail is provided to conduct similar studies in other contexts.
Confirmability	Apart from debriefing within the process evaluation team, these findings have been discussed with members of the HeLTI consortium to obtain critique and assess the resonance of these findings with the broader trial and HeLTI consortium. Feedback on the first round of focus groups was provided to participants in the second round of focus groups.

### Data analysis

A codebook thematic approach was taken to the descriptive process of data analysis.^48–50^ The analysis process began with the discussion guide, which generated some initial ideas for organisation and interpretation of the data. Based on this, plus thorough reading of the transcripts, an initial coding framework was developed by NT and NN, with input from CD. The main themes identified were perceptions of preconception and pregnancy planning, perceived impact of the trial (intervention and control arms), motivation for staying involved in the trial, agency, and suggestions for preconception services.

Once the sub-themes within these main themes were refined (by NT, NN and CD), the coding framework was then applied to the transcripts to identify relevant portions of the text that corresponded to these codes using MAXQDA data analysis software. NT and NN each separately coded four transcripts (two intervention, two control), and then met and discussed all eight of the transcripts and the codes to ensure consistency and consensus. Coded sections were then exported and summarised, and illustrative quotes were selected. In the presentation of results, where perspectives were similar between intervention and control participants, these are summarised together. They are summarised separately where perspectives differed.

### Ethical considerations

All participants gave written informed consent for their involvement in this study, and ethical approval was granted by the Human Research Ethics Committee (Medical) at the University of the Witwatersrand (reference: M190449).

## Results

### Perceptions of preconception and pregnancy planning

#### Preconception

Participants across all groups were generally unfamiliar with the term preconception health, which was evident in their verbal and non-verbal responses during the discussions. Given the original framing of the trial and intervention around young women’s health and not explicitly preconception (and becoming pregnant), this was not a surprising finding. Some participants were unaware that their existing behaviours and practices fell under the scope of preconception health until the concept was explained to them. After gaining an understanding of the term, participants acknowledged that it referred to maintaining good health, living a healthy lifestyle, and preparing their bodies for future pregnancy. Some intervention participants identified features of the health behaviour components from the *Bukhali* intervention that promoted health in the preconception period, irrespective of future pregnancy.

Participants acknowledged their limited access to knowledge regarding preconception health, both at home and at health facilities. They emphasised the need for men to be more informed about health and pregnancy-related matters. They also discussed the stigma surrounding sexual and reproductive health services, noting that this stigma affects both men and women. Illustrative quotes for this theme are provided in Table 2.

**Table 2.** Participant quotes about preconception.

Lack of familiarity with the term	*“We didn’t even think about it until you put it in the way that you did.” (I)*
	*“For me, I have never thought about it, so just like she said before, so now as you are saying it, I am going to continue because over here, obvious, I have learnt a lot about health, and making sure that my iron and my blood pressure are alright, so I am going to continue doing the things I was doing during these 18 months, but obvious, I won’t be drinking supplements, but I am going to make sure I am going to eat healthy and sleep the right amount of time, I walk and try to exercise, then make sure that I am alright mentally as well.” (I)*
Understanding of preconception	*“Yes, that is why I answered the way you asked us, yeah, but because I wanted to have a child, yeah, I stopped going out to have fun, when I had money, I would take myself to a spa, telling myself that I want to be ready for when the child is here.” (I)*
	*“For me, from now on, I will be taking supplements, and I have to take care of myself and eat healthy food, if I have baby number 2, I don’t know, but I have to get a healthy way and know that I am happy in life.” (I)*
Lack of information	*“That is the thing, with us we lack information. Where we think, we are going to get information it is actually where we are not going to get it. The easier way to get information is when you are at home, and you hear people saying telling you this and that and you take that as the correct information. The most special place where you supposed to get the information is the clinic, but you do not get it.” (C)*
Need men to be more informed	*“They don’t have the knowledge of being able to prevent as men or you can get a person pregnant which can happen once, or in seconds, and the girls forget that they can get pregnant in seconds, it’s because they don’t have knowledge, but most of them [men], they are afraid of having kids because they can’t take responsibility of that when they get someone pregnant, they all run away, they say you were sleeping with so many people, it’s not my child.”(C)*
	*“And because they [men] are afraid of going to the clinic, they are ashamed, I think the clinic for men has a stigma.” (C)*

#### Pregnancy planning

There was a discussion among participants regarding the terminology used in sexual and reproductive health, specifically between family planning and ‘preventing’. Participants stated that family planning is often perceived to be synonymous with ‘preventing’, referring to the use of contraceptives. Their comments pointed to the confusion around the implied contradiction of preventing pregnancy and planning to have children, particularly at primary healthcare facilities (clinics). There was also discussion amongst participants around the effectiveness and safety of contraceptives, and the fear of infertility at a later stage.

In addition to the confusion around terminology, participants cited negative experiences at clinics when seeking sexual and reproductive health services. Many shared that when they accessed these services, they were often not provided with essential information about contraceptives and, in some cases, encountered prejudice from healthcare providers. Some participants mentioned instances of injectable contraceptives being given to women without their knowledge and hence without their informed consent.

Perceptions around sexual and reproductive health extended beyond health facilities and into families and communities. The majority of the participants spoke about how contraceptive use was often frowned upon and viewed negatively by family and/or community members, alluding to the stigma mentioned earlier. For some family members, sending or encouraging your child to go to the clinic to seek contraceptives was indirectly seen as encouraging sexual behaviour. One community within Soweto was mentioned that viewed contraceptive use positively among young people, often motivated by the prevalence of teenage pregnancy within the community. Illustrative quotes for this theme are provided in Table 3.

**Table 3.** Participant quotes about pregnancy planning.

Terminology	*“At times it is very confusing because the first time I went to the clinic, they asked me if I was there for family planning and I said no, I do not want to have kids. They then said you need to stand on this line. I was confused until I researched it on my own because the nurse was not really telling me anything. She did not explain it to me thoroughly. She should have told me that you are literally planning to not to have a child yet. You are waiting for the right time.” (C)*
	*“To me it means physically, financially at this period of my time I am not yet ready to have a child, that is why I am preventing. They should literally call it prevention because family planning is disturbing. They should make family planning for people who actually planning to have kids, not preventing to have kids.” (C)*
Contraceptives	*“Even the implant…They say when the child gets born, they come out holding it. My friend also she gave birth to a child holding an implant. You have to always check it because it shifts.” (C)*
	*“I am also scared to go for family planning because they have already discouraged me that I will not have children. If I managed to have children, there will be with abnormalities. They also say that during their time they never used any contraceptive method. They would just fall pregnant when the time is right. They told me that this thing of planning when you want to be pregnant does not work. I was very discouraged by that.”(C)*
Experiences at health facilities	*“And then they have an attitude when they see us, the youth, the nurses get an attitude.” (I)*
	*“I wish there was something like this [Bukhali] that could help young girls, because at the clinic, it doesn’t help, yeah, at the clinic, I don’t want to lie, they don’t help, and it doesn’t cost anything.” (I)*
	*“For me, I think it is better if they asked us, you see the nurses that are at your clinic [Bukhali]. I would be happy if they were at our clinic, because they just inject people without asking questions, so I want people who are going to ask us first, if we have kids or not, not for them to just inject you with the one for 3 months without any knowledge.” (I)*
Absence of consent for contraceptives	*“So, I was also given an injection after giving birth without my consent. They put the card inside my child’s documents, and I did not notice it because no one told me about it. At home they were shouting at me asking why I not got the injection after giving birth. It was only when I took my child to the clinic for a check-up that the nurse made me aware that I missed my date.” (C)*
Family and community views about contraception	*“One thing about family planning is that, when you tell people, like in the community, right, that you are going to family planning, they are judgemental. Like you get older people saying you are killing your children, you are supposed to give birth. And then when you do get pregnant, they are judgemental and they say you like having sex and stuff.” (I)*
	*“In my community, they think it is a good thing for young people to prevent, because there are a lot of kids, or you find that at someone place, there is a lot of people and now you are pregnant and you are bringing more responsibility, you see, so they think it’s good to prevent, you see, it becomes better.” (C)*
	*“Please understand that they do not teach you about contraceptives and using condoms. My sister was like since I am dating, how about they took me to the clinic so that they can give me contraceptive pills or an injection. My aunt then refused and said that by doing that they will be sending me straight to the boys.” (C)*

### Perceived impact of the trial

#### Intervention participants

The content of the intervention material was discussed throughout the discussions with intervention participants, providing evidence that the information provided in the material has been taken on board and internalised by participants. They frequently spoke about the positive impact of the trial (which they referred to as the study) on their health, which further highlights the acceptability of the intervention. This impact related to the participants’ onsite visits (once every six months), the services being offered, their relationship with the trial staff and their Health Helpers, all of which positively influenced their health behaviour changes (e.g. physical activity, screen time, eating behaviour). Through the trial, these participants gained knowledge about their bodies, weight, eating habits, and their status for various health conditions. Participants had also noticed differences in their iron levels before and after taking the supplements, and this encouraged them to keep taking them. There was general consensus that 18 months was too short for the intervention, and that they would have preferred the intervention to continue for longer.

#### Control participants

For control participants, there was similar evidence regarding the content provided to them on life skills, and they often referred to the impact of the study being related to the life skills sessions that they had, indicating the acceptability of this component of the trial, which affirmed the decisions made around the control arm to engage participants in the trial over a lengthy period with relevant material. This impact related to the participants’ onsite visits (also once every six months), the services being offered, their relationship with the trial staff and their call centre agent, which impacted their eagerness to work on their personal goals, and seemed to build their self-awareness and self-confidence. The sessions and the material on being job ready, updating a CV and the interview tips assisted some participants with gaining employment. Control participants also found the duration of the programme too short, and wanted a focus on health for the new health programme since they only received feedback on their health during the research visits (at baseline and exit at 18 months). Some relayed how staff turnover had an impact on how they engaged with their newly allocated call centre agents and ultimately with the study, highlighting the importance of continuity. Illustrative quotes for this theme are provided in Table 4.

**Table 4.** Participant quotes about perceived impact.

Intervention	*“We are health conscious when it comes to our health because of the study.”*
	*“Yes, I have learned a lot, how I must eat, be able to exercise, the time we spend on our phones.”*
	*“The thing that helped me is the exercises and eating healthy, before I didn’t know that.”*
	*“The study helped even with the supplements because some found out they have low iron, so they could take the supplements and be alright.”*
	*“Yeah, it was alright, and the supplements were good as well, because they would tell us how to eat, because a lot of us were not eating spinach which is something good for our bodies, and liver as well.”*
Control	*“The study made me feel, I don’t know, like I am able to control my life.”*
	*“Coming here has really helped me a lot. I did not know that volunteering can take you somewhere in life. I always had that perception of saying we all want money, there is no way that you can tell me that I should go and do some work for free.”*
	*“I now have confidence, before I didn’t have confidence because before I had a problem with an abusive baby daddy and I didn’t know what to do with it, I didn’t know whether to get him arrested or if I should hit him back. So now I have the confidence because I was able to make a decision about what to do with the situation, and so I decided to leave the relationship and raise the baby on my own.”*
	*“For me it was to do a curriculum vitae and I did it and got the job that I wanted.”*
	*“I think two years ‘cause you see the more the period is long, the more I would also see other things differently.”*

### Motivation for staying in the trial

In all the focus groups, reimbursement was the leading motivation for staying involved in the trial. Participants stated that the reimbursement was not only used for transportation, but also for catering for their and their families’ needs due to their socioeconomic challenges. This was mostly the case for participants who were unemployed.

Most of the participants relayed how they later stayed in the trial because of the knowledge and new information that they gained, which is further support of the intervention’s acceptability. Intervention participants went as far as stating that the health feedback was an eye opener for them since they gained knowledge on their health behaviour, and how they could make changes. For control participants, the health feedback from their baseline research visit, and the information sheet (provided to them as part of the consenting process) provided some motivation for health behaviour change. Illustrative quotes for this theme are provided in Table 5.

**Table 5.** Participant quotes about motivation.

Financial motivation	*“Money, because sometimes you get it when you really don’t have anything, and when she calls you and books you, you are at least able to buy mielie meal at home and the rest is a bonus.” (C)*
	*“To us as teenagers, you just want to buy toiletries. If you have it, you add on top of that. And most of us don’t have it so it makes a lot of difference.” (C)*
	*“I will not lie to you, at first for me it was about of the money because I was just sitting at home and doing nothing. Those few hours of coming out of home and coming here made a difference a lot. Hence I have said that I am one person who does not get sick.” (C)*
	*“And then…then the study has helped us, you can see what’s happening in our country, in terms of unemployment…, like the unemployment rate. We end up having done some mistakes at home, so when we receive a call from you guys telling us that you’ll fetch us, and so on and so on. It gives us that…I won’t say we are happy because of the money that we get when we leave, but it gives us joy.” (I)*
Learning motivation	*“I found this study very interesting. My group was a bit different from the other groups because I know the other groups were taking samples and everything but where I went, we would go and they would ask us questions and give us information. They would send us links on how to conduct an interview and they would tell us how much the drivers licence is. Basic things that people never knew about. So yes, we got a lot of information. Whenever you came to the study you would go back filled with information that you never knew. That was my experience.” (C)*
	*“So yesterday while I was here, they were asking us about the food that we eat in the restaurants that we often visit and how often do we visit them. So, I have notice that I do not eat healthy, it is only once in a while. Sometimes I would visit my boyfriend in the North for one or two weeks and we would eat takeaways every day. We hardly eat healthy food and that is when I notice that I need to actually start eating healthier. I am also thinking of joining a gym because I want to have a flat stomach. I cannot have a flat stomach while eating chicken maybe five times. Yesterday I was eating KFC and on Wednesday I was having Chicken Licken. I would ask myself when did I eat vegetables or healthy this week? The only thing I could remember is that I had a tomato.” (C)*
	*“I think I learned to always take care of myself because the first time I came here, they gave me a referral letter to go to the clinic because my high blood was high, so they helped me because I now get it checked every month and now it is good.” (C)*
	*“The first time I came here I lacked iron and I did not have any knowledge about it. At school we were donating blood but they would tell me that I did not qualify and I did not take that into consideration until I came here. They advised me about food that I needed to eat such as fruits and vegetables in order to help increase my iron level.” (C)*

### Agency

Based on previous qualitative work in the *Bukhali* process evaluation, and the relevance of agency to reproductive health, we were interested in understanding participants’ sense of agency as a result of being involved in the trial, both in the intervention and control arms, as another indicator of acceptability. While the term ‘agency’ was not specifically used (given that it may not translate well in local languages), participants were rather asked about their feeling of control in their life, how they feel about making decisions, and how these have changed over the course of being in the trial. For both groups, it was evident that for many participants, their involvement in the trial (intervention and control arms) had contributed to their sense of agency.

Participants linked agency with setting boundaries, making better decisions, and choosing how they would associate with their friends. Their ability to make decisions for themselves gave them a sense of hope in their ability to be successful in life; it boosted their confidence and brought them happiness. However, there were participants who felt that they did not have complete control over their lives, which they attributed to their age and the acknowledgment of decision-making being a process. For intervention participants, decision-making involved making decisions about their health, such as changing their eating habits.

Related to decision making, goal setting was described in a positive light, as it gave participants a sense of hope and something to look forward to. Setting a goal allowed them to plan the things they would like to achieve. Conversely, failing to attain the goals, especially for those whose goals were employment, brought about negative feelings. Illustrative quotes for this theme are provided in Table 6.

**Table 6.** Participant quotes about agency.

Increased agency	*“I am happy as well, yeah, with my health, that I am able to take my own decisions.” (I)*
	*“Like right now, we are able to talk with our Health Helpers and they advise us about life, you don’t just follow what the other people are doing, like peer pressure, you can think for yourself.” (I)*
	*“Yes, I can now take decisions for myself alone without anyone else, and when I do decide, I can think about myself so that no matter what happens, I am the one who is going to have to stand up for myself.” (I)*
	*“I feel in control because when I came here, I was still going to school, and I was dealing with a lot of things, so they helped me with my mental health issues. And also, I had a high BP all the time but right now I am able to go and get myself checked because every time we attended a session; we would have a health check done on us.” (C)*
Decision making	*“I can now make certain decisions for myself, I am still learning, it is not like I have everything under control. I am still in a process of learning everything else but so far this study has given me a start-up.” (C)*
Goal setting	*“I think it’s very important to set goals because when you are working on something you are able to push yourself, like you do more.” (C)*
	*“Life is difficult and we are going through a lot of things, however goal setting gives you something to look forward to.” (I)*
	*“I feel like goal setting is a good thing as you get to plan the things you want to achieve in life step by step.” (I)*
	*“Goal setting is depressing because you can see that you are trying, like really trying to get a job but you are being unsuccessful, that is the most depressing thing because it makes one lose hope.” (I)*

### Suggestions for preconception services

**Figure 1.**
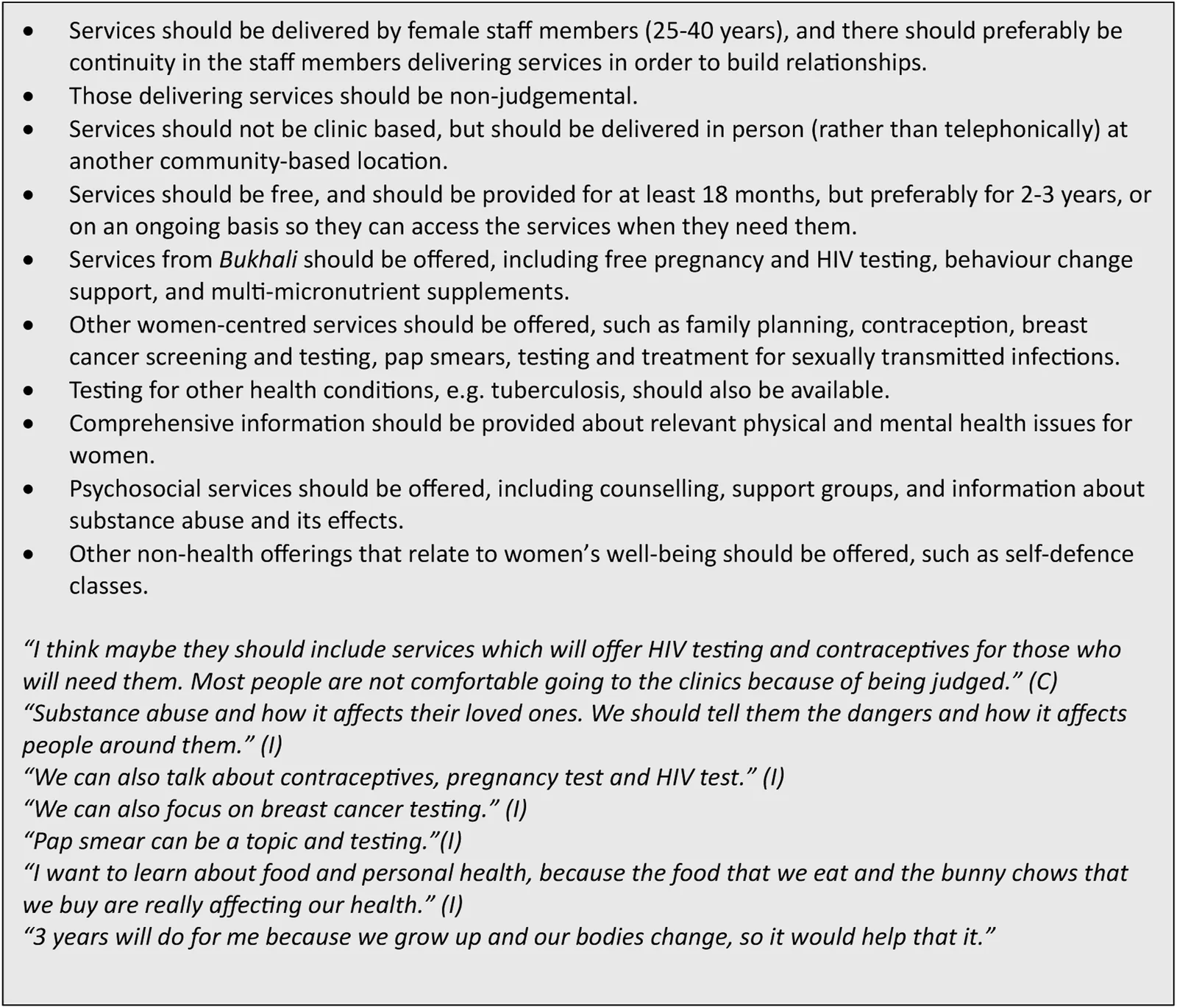
Participants’ suggestions for preconception services.

Suggestions made by participants for preconception services that could be implemented in the future have been summarised in Figure 1, with illustrative quotes provided. Key suggestions mentioned related to learning, which seemed to involve both receiving information and talking, about a holistic range of physical and mental health topics. Linked to this, testing for various health conditions was often mentioned, which can be taken as an indication of participants’ desire to learn about their health status, and be better informed about their health. Comments regarding the length of time that these services should be offered imply that these services are less about a once-off impartation of knowledge, but rather about a relationship with these services that acknowledges them as agents in their health journey.

## Discussion

The process evaluation of the *Bukhali* preconception phase of the trial highlighted that the trial was viewed as acceptable by women participating in both the intervention and control arms of the trial. This acceptability included the content provided to both arms of the trial, as well as the perceived impact of the trial (for both arms), participants’ motivation to stay involved in the trial, and the sense of agency that women develop through their involvement. These findings reinforce previous *Bukhali* process evaluation findings^36,40^ regarding the value that participants placed on what they had learnt from the trial, the benefits of the trial for their physical and mental health and well-being, their shift to intrinsic motivation over time, and a growing sense of autonomy and agency. Similar to other qualitative work conducted with *Bukhali* participants relating to agency,^51^ participants’ sense of agency is supported by previous research on women’s agency, particularly regarding their health, linking agency to goal setting, choices and decisions, negotiating and navigating agency over time,^52–54^ and acknowledging the complexity of agency for women in vulnerable settings, such to Soweto.^53,55^

Although women’s low levels of awareness around preconception align with previous studies in South Africa^17–19^ and elsewhere in Africa,^11,16^ the findings of this study indicate that a health promotion intervention focussed on young women can improve health literacy over an 18-month period. However, preconception remains an unfamiliar term in this target group, which is not unexpected given that this has been commented on more generally, and relates to literacy challenges.^22^ Related to this, terms such as pregnancy planning, family planning, and ‘preventing’ only add to the confusion around the framing of health promotion for young women who are not pregnant in a context such as Soweto. Future process evaluation research could explore differences in participants’ understanding of these concepts, and investigate in more detail the factors that facilitate or hinder this understanding amongst individuals within this context.

While literature has called for a lifecourse approach to women’s health,^1,56^ this study illustrates that framing interventions solely around fertility may alienate or confuse participants. Instead, moving beyond biomedical framing and adopting a biosocial perspective that acknowledges the complex interplay between contexts, social relationships, and biological phenomena,^57^ preconception health should be positioned as a pathway to general well-being and empowerment increases resonance and acceptability. This aligns with the formative work around the framing of the *Bukhali* trial and intervention. From a policy and practice perspective, framing young women’s health around physical and mental health issues that are relevant to them would be far more effective than framing it around preconception, at least in this context. Although intervention participants did not have any exposure to the life skills material provided to control participants, the acceptability of the control component suggests that life skills could be included in the offering as part of these services. Future research could explore the combined feasibility, acceptability, and efficacy of health plus life skills in contexts where socioeconomic challenges are pervasive.

Given Ukoha and Mtshali’s findings in South Africa,^17^ it would be helpful to explore perceptions of the term ‘reproductive life plan’ with young women in Soweto, given their specific request for more information about sexual and reproductive health, especially contraception. This aligns with recommendations for a holistic model that integrates reproductive life planning, contraception, and preconception care – albeit based on evidence from high-income country settings.^6^ In the South African context, it would be helpful to understand, firstly, whether such a model accommodates women’s health more generally for young women, acknowledging the importance of sexual and reproductive health, or whether it perpetuates the conflation of women’s health and maternal health.^21^ Secondly, how do young women respond to the notion of planning within the context of their lived experiences? Planning has connotations of intentionality and choice, and these may not be accessible to all women,^22^ including in the South African context. The findings of this study suggest that planning (incorporating goal setting and a sense of agency) is a key mechanism of change and aligns with calls for rights-based approaches to health programming that go beyond biomedical metrics.^22^

Moreover, in contrast to vertical, time-limited programs, this study shows that long-term, relational approaches (aligned with continuity of care and health literacy empowerment) drive behaviour change more sustainably. It supports findings from adolescent and youth-friendly services literature that trust, communication, and sustained engagement are essential.^58^ Indeed, the preference of participants for a longer length of time for a preconception services (or intervention), which makes sense considering that the perceived impact of the trial takes time. In support of the need for time, participants’ positive feedback about goal setting is in contrast to earlier process evaluation findings with participants at the start of their involvement in the trial, where planning and goal setting were identified as a challenge, and viewed through a trauma-informed perspective that is highly relevant in the Soweto context.^35^ While learning, skills development, health benefits, motivational shifts, and increases in agency can take place over the short-term (in ideal circumstances), these are more likely to take longer to be optimised in challenging contexts like Soweto. However, a critical question remains: given the importance of financial incentives for participants (reported elsewhere as a significant driver of retention for the trial^40^), will young women access these services if they are not paid? Financial reimbursement was a strong initial motivator for participation, and should be factored into future programme design, particularly in the early phases of programme implementation. However, over time, knowledge gain, emotional support, and personal transformation took precedence. This evolution in motivation suggests that even in socioeconomically constrained settings, well-designed interventions can engage intrinsic motivators for sustained behaviour change.

Related to the length of time for preconception services, the findings of this study have underscored previous process evaluation findings^36,40,42^ that *who* delivers such services (e.g. female, younger in age), and *how* they are delivered are critical. It is clear from these and previous findings^40,59^ that the delivery agents should not be nurses in clinics, given how participants feel about their treatment in these settings by these individuals. Rather, participants have made it clear (in this and previous work^40^) that preconception services should be delivered in a context that is autonomy-supportive and free from judgement and stigma. This aligns with recommendations to incorporate a gender rights and equity perspective, and a continuum of care approach across stages of the life course.^22^ Previous findings indicate that the context of the *Bukhali* trial has provided this safe space that participants need.^40^

This study’s findings imply that this context should provide a conducive environment for learning and development over time, which includes the types of relationships that would support this. Previous findings indicate that the *Bukhali* trial staff have been able to provide this type of supportive relationship,^36,40,42^ and this can serve as a model for how such community-based services can in turn support and complement the services offered at health facilities. This not only provides a service that is acceptable to young women, but also contributes to a reduction in the burden on already over-burdened healthcare services. Such a community-based approach aligns with recommendations for a holistic model mentioned earlier, although the recommended approach proposes intervention at multiple levels (e.g. school-based education, social media, national campaigns) that links to primary healthcare services.^6^ While intervention at these levels may have value in the South African context, they were not specifically suggested by the participants of this study, but could be explored in future research.

### Limitations

The focus on a particular intervention context could be viewed as a potential limitation of this study, as well as the sampling of women who remained in the trial. Achieving data saturation was not an explicit aim of this study. Women who had dropped out of the trial or were lost to follow-up may well have had different and less positive experiences of the trial, and thus less positive opinions on the acceptability of the trial. Recruiting these individuals is inherently difficult in these types of qualitative studies (e.g. women who were not contactable for the trial remain uncontactable for focus groups), however view of such individuals have been reported on elsewhere for this trial,^46^ including in relation to retention in the trial.^40^ Given these challenges around recruitment, it was difficult to pilot-test the interview guide, which is acknowledged as a limitation. In addition, the tension held between somewhat of a reliance on preconception as a term and the interrogation of this term could also be viewed as a limitation. However, acknowledging the contextual nuances of this term was intended to mitigate this limitation.

A strength of this study is that it provides contextually rich insights into the acceptability of a preconception trial (and intervention), from the perspectives of participants. While qualitative findings are not intended to be generalisable, the findings of this study provide pragmatic suggestions on preconception services and interventions that could be implemented in contexts similar to Soweto. These could include other urban and rural settings within South Africa, as well as in other low- and middle-income countries where similar socioeconomic challenges exist, and where health services are not adequately meeting the needs of women in these contexts. Specifically, the suggestions for preconception services made by participants in this study could be relevant and appropriately adapted to these other contexts, e.g. continuity of care that is non-judgemental, financially accessible and some free services (e.g. pregnancy and HIV testing) available over time, as well as the availability of women-centred services, psychosocial and other non-health offerings that meet local needs of women.

### Conclusion

In conclusion, the Bukhali process evaluation provides evidence that intervention is perceived as acceptable and impactful. Furthermore, these findings add critical nuance to the field of preconception and reproductive health by: (i) reframing preconception health as holistic, not just fertility-linked; (ii) stressing the importance of relational and continuity-based care; (iii) bridging structural barriers through contextualised participant-centric design; and that (iv) agency is a measurable and meaningful outcome in preconception interventions. By elevating women’s agency, understanding of health, and trust in health systems, this model challenges traditional, biomedical-focused frameworks and calls for reimagining of preconception health as an empowering, inclusive, and continuous process.

## Supplemental Material

Supplemental Material - Participants’ perceptions of the preconception phase of the *Bukhali* trial for young women in Soweto, South Africa: A qualitative study Supplemental Material for Participants’ perceptions of the preconception phase of the *Bukhali* trial for young women in Soweto, South Africa: A qualitative study by C. E. Draper, N. Tshetu, N. Nkosi, S. J. Lye, S. A. Norris in Women’s Health.

Supplemental Material - Participants’ perceptions of the preconception phase of the *Bukhali* trial for young women in Soweto, South Africa: A qualitative study Supplemental Material for Participants’ perceptions of the preconception phase of the *Bukhali* trial for young women in Soweto, South Africa: A qualitative study by C. E. Draper, N. Tshetu, N. Nkosi, S. J. Lye, S. A. Norris in Women’s Health.

Supplemental Material - Participants’ perceptions of the preconception phase of the *Bukhali* trial for young women in Soweto, South Africa: A qualitative study Supplemental Material for Participants’ perceptions of the preconception phase of the *Bukhali* trial for young women in Soweto, South Africa: A qualitative study by C. E. Draper, N. Tshetu, N. Nkosi, S. J. Lye, S. A. Norris in Women’s Health.

## Data Availability

Due to the risk of identifying participants, the authors do not have ethical approval to share the qualitative data used for this paper.*
