# Supplementary material for: Participants’ perceptions of the preconception phase of the Bukhali trial for young women in Soweto, South Africa: A qualitative study
Source: Womens Health (Lond). 2026 Aug 25;22:17455057261469051. doi: 10.1177/17455057261469051 (PMC13519232; doi:10.1177/17455057261469051)
Supplement: Supplementary file 1 [file sj-pdf-1-whe-10.1177_17455057261469051.pdf]

# Focus group guide questions: Intervention and control participants

**Round 1:**

- What has it been like to be part of *Bukhali* for you?
    - What did you find most impactful about the study?
    - How would you describe your relationship with your HH / CCA?
    - What do you think about the material covered in the study?
    - What would you say you have learned about yourself from being part of *Bukhali*?
- What made you stay involved in *Bukhali*?
- Agency:
    - How in control do you feel of your life, and how has this changed over the time you have been part of *Bukhali*?
    - How do you feel about making decisions in your life, about their health etc., and how has this changed over the time you have been part of *Bukhali*?
    - Intervention: probe about changes in health behaviours (physical activity, sleep, sedentary behaviour, screen time, eating/drinking habits) in relation to agency
    - Control: probe about life skills in relation to agency
- Preconception health
    - What does 'preconception health' mean to you?
    - *Definition of preconception to share: 'Preconception health refers to the health of people during their reproductive years, or the years they can have a child. It focuses on taking steps now to protect the health of a baby they might have some time in the future'*
- How should the topic of preconception health be presented to young women (and men) in your community?
- Family planning:
    - What does this term mean to you?
    - How does this family planning make you feel?
    - What are your beliefs around family planning?
    - What social / cultural / religious influences have affected these beliefs?
    - What are your community's beliefs about young women using family planning services?
- Pregnancy planning:
    - What do you think about pregnancy planning (i.e. deciding when to have a baby)?
    - How do women think about this in your community?
    - How do men think about this in your community?
    - Imagine you were currently hoping to become pregnant, what would be the most important factors for you to have in place before the pregnancy?
        - Where does your mental or physical health come on your list of priorities?
        - Where do other factors, such as money, living conditions, relationships and family, work/education, climate change (+ other factors mentioned in the discussion) come?
        - How did you decide which factors you put at the top of your list?
        - Why are the factors at the top of your list more important than others?

    - If you were currently hoping to become pregnant, are there any health-related factors you would want to change to increase the chances of a healthy pregnancy?
    - What preconception care services would you like to see in your community that you would access if available?
    - How should we approach this topic in your community? Should we address this at all?
- How have your choices about having a baby changed over the time you have been involved in *Bukhali*?
- What has influenced your choice to not have a baby?

**Round 2:**

- Provide summary of key findings from previous FGs (intervention and control separately) in ~10 minutes.
- Given the challenges young women face in Soweto, what would be their recommendations be for a health programme targeting young women, considering (and probing) the following:
    - Duration: how long should the programme be?
    - Intensity: how often should the programme sessions be?
    - Who should deliver the sessions, and what are their reasons for this?
    - Content: what should the programme cover? *(provide short summary of previous topics they mentioned)*
- What else do they think is needed to get them to improve their health?
- How do they feel about goal setting for their health, and for other things (e.g. education employment?
