# Supplementary material for: Participants’ perceptions of the preconception phase of the Bukhali trial for young women in Soweto, South Africa: A qualitative study
Source: Womens Health (Lond). 2026 Aug 25;22:17455057261469051. doi: 10.1177/17455057261469051 (PMC13519232; doi:10.1177/17455057261469051)
Supplement: Supplementary file 2 [file sj-pdf-2-whe-10.1177_17455057261469051.pdf]

Catherine E. Draper is a mid-career academic. She identifies as white and English-speaking, and was born and resides in South Africa. She trained in psychology and public health, with a focus on qualitative research methods. She has been doing cross-cultural, community-based research for just over 15 years in low-income urban and rural settings in South Africa. Her current work uses mixed methods, and includes work in implementation science and qualitative methods. She oversees the intervention, control and process evaluation components of the *Bukhali* trial.

Nosibusiso Tshetu is a Master of Arts in Women's and Gender Studies candidate. She is an African who identifies as a woman. She was born in South Africa where she still resides to this day. She holds a Bachelor of Arts Honours degree in Women's and Gender Studies and a Bachelor of Arts degree in Psychology, Ethics, and Women's and Gender Studies. Her research interests have been in young women's experiences of pregnancy and motherhood, and early childhood development. She is the Project Coordinator for the process evaluation of the *Bukhali* trial.

Nokuthula Nkosi is a Research Assistant with 4 years of experience in the field. She identifies as an African-Zulu speaking and resides in Soweto-Diepkloof, which makes her well positioned in identifying with the cohort. She trained as a Social Worker before moving into research. She largely focuses on qualitative research which was rooted during her undergraduate and postgraduate studies at the University of the Witwatersrand. She holds a Masters in Social Work (Occupational Social Work) and Masters by Dissertation (Development Studies). She is currently working as a Research Assistant for the process evaluation of the *Bukhali* trial.

Stephen J Lye is is Professor in the Departments of Obstetrics & Gynaecology, Physiology and Medicine at the University of Toronto. He identifies as white and English-speaking, and was born in the UK and resides in Canada. He leads the Alliance for Human Development, a transdisciplinary initiative that seeks to improve the health and well-being of all children, everywhere, by focusing on optimizing their development in early life. He is a Co-Principal Investigator on the international Healthy Lifestyles Trajectory Initiative, specifically HeLTI SA (*Bukhali).*

Shane A Norris is a Professor of Global Health at the University of Southampton (UK) and Research Professor at the University of the Witwatersrand, Johannesburg, South Africa. He identifies as white and English-speaking, and was born in the South Africa and resides in the UK. He is an epidemiologist and is the Director of the South African Medical Research Council's Developmental Pathways for Health Research Unit. He is a Co-Principal Investigator on the international Healthy Lifestyles Trajectory Initiative, specifically HeLTI SA (*Bukhali).*
